# Vaginal palpation versus transabdominal ultrasound in the comprehension of pelvic floor muscle contraction after vaginal delivery: a randomised controlled trial

**DOI:** 10.1186/s12905-021-01203-w

**Published:** 2021-02-06

**Authors:** Mayumi Ikeda, Akiko Mori

**Affiliations:** 1 Graduate Course of Midwifery, Teikyo University, 2-11-1 Kaga, Itabashi-ku, Tokyo, Japan; 2 Department of Nursing, Shonan Kamakura University of Medical Sciences School of Nursing, 1195-3 Yamazaki, Kamakura-shi, Kanagawa Japan

**Keywords:** Pelvic floor muscle contraction, Vaginal palpation, Transabdominal ultrasound, Vaginal delivery, Randomised controlled trial, Bladder base, Postpartum, Urinary incontinence, Perineal injury

## Abstract

**Background:**

Pelvic floor muscles support the pelvic organs and control voiding. The first choice in the repair of pelvic floor function that is damaged during pregnancy and delivery is pelvic floor muscle training, which involves repeated muscle relaxation and contraction. However, as muscle contractions cannot be visualised, it is difficult to assess whether patients understand how to contract them. Therefore, we assessed patients’ comprehension of pelvic floor muscle contraction by comparing two teaching methods, vaginal palpation and transabdominal ultrasound, following vaginal delivery. We hypothesised that vaginal palpation is better than transabdominal ultrasound in this regard.

**Methods:**

This randomised controlled trial conducted in facilities in Tokyo, Japan between July 2018 and January 2019 included women aged ≥ 20 years at 4–6 weeks after vaginal delivery. The randomisation involved website-based centralised allocation. The primary outcome was a change in bladder base displacement during pelvic floor muscle contraction before and after training, which was measured using transabdominal ultrasound. Participants performed three contractions for 3 s, and the mean value was used for statistical analysis. The secondary outcome was a change in understanding the contraction before and after training, which was measured using a five-point Likert scale questionnaire. Outcomes were analysed using Welch’s t-test.

**Results:**

Sixty-five participants were randomly allocated to the vaginal palpation group (n = 32) and transabdominal ultrasound group (n = 33). Baseline characteristics were similar between the groups. Changes in bladder base displacement were not significantly different between the groups (*p* = 0.181). Within-group analyses showed that bladder base displacement was large in both groups after the respective intervention. There were no significant differences in any of the outcomes between the two groups before and after the intervention.

**Conclusions:**

Vaginal palpation and transabdominal ultrasound might be useful for comprehending pelvic floor muscle contraction after vaginal delivery.

*Trial registration*: UMIN 000032304. Registered 18 April 2018, https://upload.umin.ac.jp/cgi-open-bin/icdr_e/ctr_view.cgi?recptno=R000036820.

## Background

Vaginal delivery carries the highest risk for lower urinary tract symptoms; the incidence of such symptoms is 6.1 times higher in women who underwent vaginal delivery than in those who underwent caesarean section [1]. Pelvic floor weakness and urinary incontinence are important issues in women’s health. Vaginal delivery is an independent risk factor for damage to the pelvic floor muscles (PFMs) [2], and pelvic floor distensibility may cause pelvic floor dysfunction, such as urinary incontinence and pelvic organ prolapse, later in life irrespective of the delivery mode [3]. PFM training is commonly recommended during pregnancy and postpartum period for the prevention and treatment of incontinence [4]. Recent studies have shown that PFM training is effective for the treatment of genitourinary syndrome of menopause as well as reducing its symptoms and signs and its effects on activities of daily living, quality of life, and sexual function [5]. It has been suggested that PFM training improves blood flow and elasticity of the vulvovaginal tissue [6]. Therefore, education on how to correctly contract PFMs and increase their strength, during and after pregnancy, may also contribute to postmenopausal women’s health. As many women are not aware of the preventive measures and treatment options, it would be beneficial to raise such awareness and provide the required care by midwives [7].

In Japan, education on PFM training after delivery commonly includes only oral teaching by midwives using leaflets. However, since PFM contraction cannot be visualised, it is difficult for women to comprehend how to contract and relax based on oral teaching alone. Furthermore, women do not generally know how to perform PFM contraction and therefore are unsure if they are performing contractions correctly during PFM training [8]. Therefore, it is necessary to establish a teaching method that enables such learning.

Vaginal palpation (VP) has been shown to be important in teaching how to perform PFM contraction correctly [9]. It has been reported to be a superior method compared to sonography for measuring indices of contractile function [10] and the gold standard to assess PFM contraction ability [11].

Recently, ultrasound was introduced in clinical practice as a new method to assess correct PFM contraction [12]. Both transperineal and transabdominal ultrasound (TAU) were shown to be reliable in measuring movement during PFM contraction [13]. Especially, TAU is non-invasive, allows visualisation, provides immediate visual feedback, and is easy to apply [14]. TAU imaging is used to assess PFM contraction by observing the movement of the bladder base as a surrogate marker for PFM activity. Bladder base displacement because of voluntary PFM contraction has been corroborated by previous findings [15].

Postpartum women are expected to have reduced pelvic floor function; therefore, we predicted that VP, as a direct tactile evaluation, would an easier method in understanding PFM contraction than TAU. The aim of this study was to assess the changes in comprehension of PFM contraction by comparing the two teaching methods, VP and TAU, following vaginal delivery. We hypothesised that VP is better suited than TAU for understanding PFM contraction.

## Methods

### Study design and participants

This two-arm randomised controlled trial was conducted in three facilities in Tokyo, Japan, in women who gave birth in primary facilities, such as midwifery home and gynaecology clinic. Data were collected between July 2018 and January 2019. The eligibility criteria were as follows: (1) age ≧ 20 years; (2) vaginal delivery; and (3) 4–6 weeks after term delivery. Women were excluded from the study if (1) they had previously received guidance for PFM contraction via VP or TAU; (2) they deviated from a normal status of obstetrics and urology; (3) they had perineal pain or numbness at the time of intervention; (4) they had urinary nerve damage; and (5) they were not able to read and write in Japanese.

This trial was registered with the UMIN Clinical Trials Registry (registration no. UMIN000032304).

### Randomisation

The participants were assigned to either the VP or TAU group. The randomisation involved centralised allocation managed by a web-based randomisation system with permuted blocks of four. Due to the nature of the intervention, masking of the intervener and participants was not possible, and the data were not masked by assignment during the analysis.

### Procedures

The intervention was performed by the intervener (midwife) alone, who implemented either the technique of VP by touching the PFMs in the VP group or TAU by showing an extracted image in the TAU group. The details of each teaching protocol are as follows.

*VP group*: The intervener slowly inserted two fingers of the right hand into the participant’s vagina and instructed the participant to lift and squeeze around the fingers. The intervener then provided feedback to the participant regarding the contraction on VP. Using the left hand, the intervener held two fingers of the participant’s right hand similar to those inserted into the vagina for palpation. The intervener alternately squeezed and relaxed the participant’s right hand in conjunction with the intravaginal contraction in order to provide tactile feedback to the patient.

*TAU group:* The intervener placed the ultrasound probe in the sagittal plane just above the pubic bone and instructed the participant to lift and squeeze the pelvic floor toward the head, while showing the extracted bladder base images on the ultrasound screen. The intervener taught the participants how to contract the PFMs by showing them the TAU images. The intervener provided feedback to the participants about the contractions using ultrasound images of the elevation of the bladder base during PFM contraction and the descent of the bladder base during relaxation.

To ensure uniformity and objectivity of each intervention, the teaching instructions were integrated according to specific protocols. However, the instructions that were similar in both groups were as follows:

1. The intervention was performed in a private room and at least 1 h after urination. The participants lay in a crook-lying position (supine position with hips and knees flexed) [16] with their soles flat on the same level of the bed and a pillow underneath their head.
2. The method of PFM contraction was explained while explaining the anatomy of PFMs using a diagram.
3. The participants practiced 10 fast and 10 endurance contractions without contracting the abdominal muscles while maintaining normal breathing.
4. The participants practiced for 10 min.

### Study outcomes

**Fig. 1:**
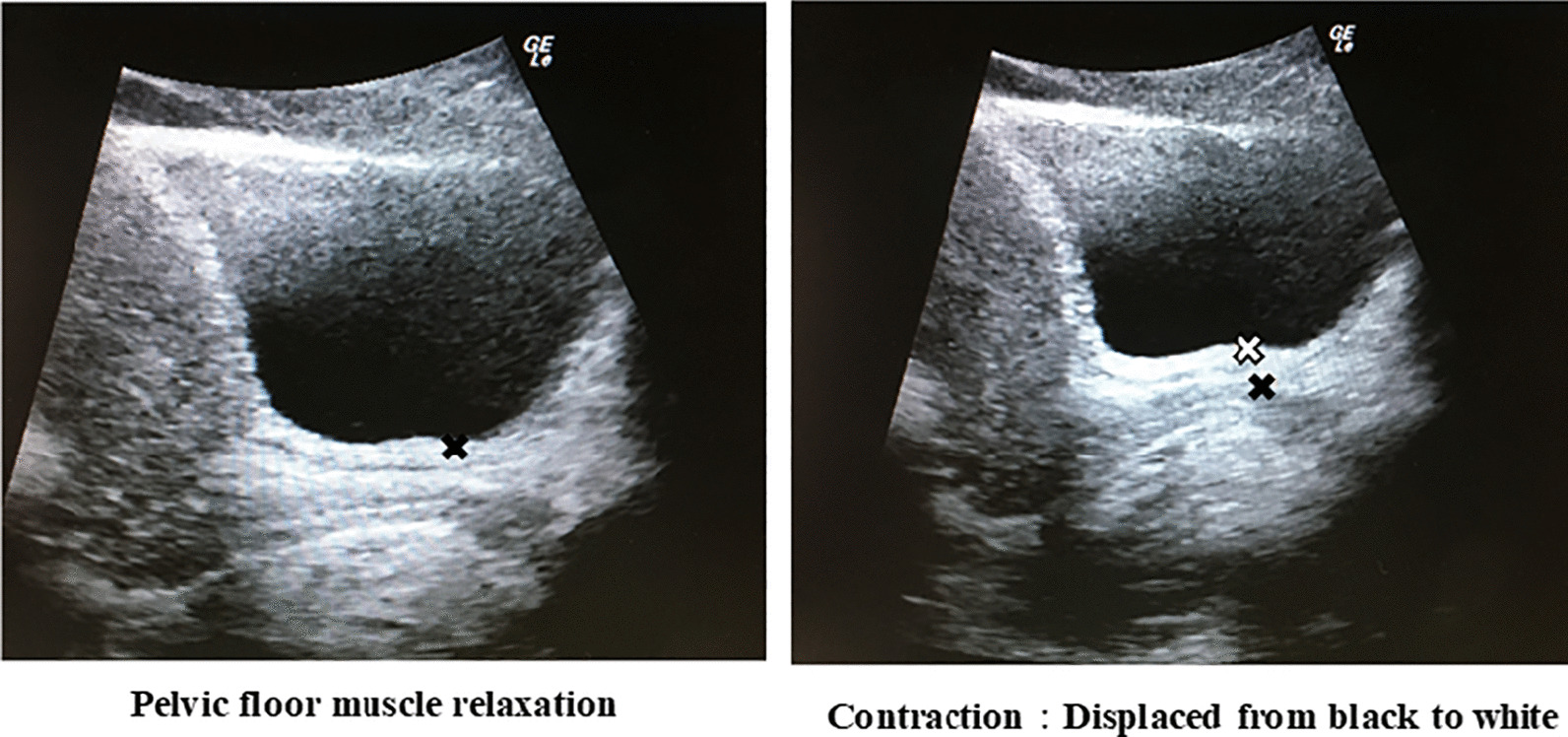
Displacement of the bladder base during pelvic floor muscle contraction

Changes in the pre- and post-intervention comprehension of PFM contraction were assessed by changes in the displacement of the bladder base during contraction and changes in understanding the contraction. Changes in bladder base displacement were measured using TAU pre-intervention, and post-intervention changes were measured similarly after a 5-min break. The measurement was performed without showing the ultrasound screen to the participants to avoid any visual feedback effect. The TAU probe was placed in the mid-sagittal plane immediately supra-pubically on the lower abdomen [11]. The measurement point was a clearly defined edge of maximum displacement of the fascia between the interureteric ridges in the bladder base and bladder neck [17, 18], and the distance between PFM relaxation and contraction was measured in mm using an on-screen calliper. The participants performed three repetitions of maximum voluntary contractions for 3 s with 10 s of relaxation between each contraction, and the mean value was used in the statistical analysis [19]. TAU is valid and reliable in assessing PFM function, and different bladder volumes do not influence the displacement measures; therefore, a strict bladder filling protocol was not necessary [20]. TAU was performed using LOGIQeV2 (GE Healthcare Japan Corp, Tokyo, Japan) with a 3.5–5.5-MHz curved array transducer. A representative image of the displacement of the bladder base during PFM contraction is shown in Fig. 1.

Pre- and post-intervention changes in understanding PFM contractions were assessed using a two-item questionnaire, which included the questions ‘Do you know where to contract in the body?’ and ‘Do you understand the sensation of contracting the pelvic floor muscles?’, as previously reported [21]. The questionnaire used a five-point Likert scale (1–5 points), and a higher score indicated better understanding.

Prior to the intervention, urinary incontinence was assessed using the Japanese version of the International Consultation on Incontinence Questionnaire-Short Form (ICIQ-SF), which was created through the process of linguistic validity verification, and its reliability and validity have been previously verified [22]. We have obtained permission to use the Japanese version of ICIQ-SF. We also collected information on the frequency of PFM training and demographic information about the participants.

### Statistical analyses

Statistical analyses were performed using SPSS Statistics v24 for Windows (IBM Inc., Armonk, NY, USA). Comparisons of the demographic data, ICIQ-SF scores, and frequency of PFM training were performed using the chi-square test and t-test. Changes in the comprehension of PFM contraction between the groups were compared using the Welch’s t-test.

### Sample size

The sample size was calculated based on a previous randomised controlled study that verified the effects of PFM training in women with pelvic organ prolapse [23]. In that study, the intervention group received individual training by a physiotherapist for 6 months, and the control group received guidance as usual; the resting position of the bladder increased by a mean of 4.2 mm [95% confidence interval (CI), 2.8–5.6] in the intervention group and − 0.1 mm (CI, − 1.9 to 1.6) in the control group. Therefore, for an effect size of 0.75, power of 80%, and significance level of 0.05, the estimated sample size was 28 women in each group.

## Results

### Participant inclusion

**Fig. 2:**
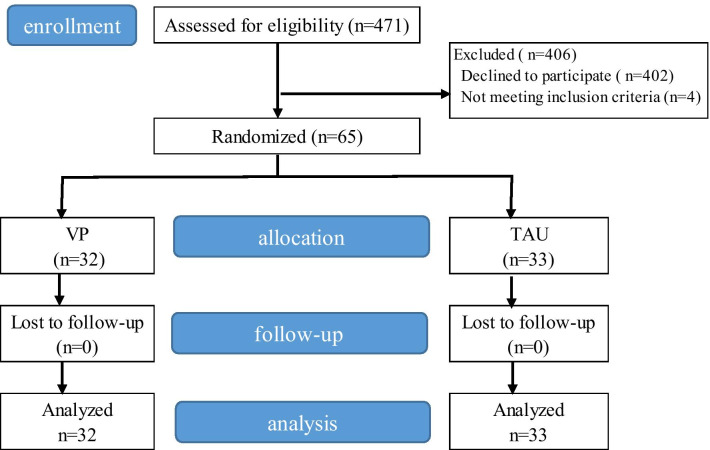
Inclusion of the participants in the study. *VP* vaginal palpation, *TAU* transabdominal ultrasound

For this study, 471 eligible postpartum women were identified between July 2018 and January 2019. When the sample size was reached, the recruitment was stopped. Four women were excluded before randomisation because they were not within the 4–6 weeks postpartum period, and 402 women refused the invitation to participate. Therefore, a total of 65 women participated in the study; 32 were allocated to the VP group and 33 to the TAU group. After random assignment, there was no dropout until the end of the study, and all of the 65 participants were included in the outcome analysis (Fig. 2).

### Baseline characteristics

Baseline data comparison revealed no differences between the two groups in all items, including the demographic data, frequency of PFM training, status of urinary incontinence, and ICIQ-SF score (Table 1).

**Table 1:** Baseline characteristics of participants

Characteristics		VP n = 32	TAU n = 33
Postpartum days	M (SD)	36.8 (5.4)	37.2 (5.4)
Age (years)	M (SD)	34.9 (3.8)	33.2 (4.1)
Body mass index before pregnancy	M (SD)	21.0 (2.8)	20.9 (2.4)
Duration of labor (min)	M (SD)	420.6 (247.1)	353.9 (261.8)
Birth weight (grams)	M (SD)	3142.5 (293.8)	3028.3 (326.0)
Parity			
Primipara	n (%)	7 (21.9)	9 (27.3)
Multipara	n (%)	25 (78.1)	24 (72.7)
Type of delivery			
Normal	n (%)	28 (87.6)	30 (90.9)
Vacuum extraction	n (%)	2 (6.3)	1 (3.0)
Epidural birth	n (%)	2 (6.3)	2 (6.1)
Perineal condition			
Episiotomy	n (%)	1 (3.1)	4 (12.1)
Laceration, first degree	n (%)	10 (31.2)	6 (18.2)
Laceration, second degree	n (%)	4 (12.5)	8 (24.2)
Episiotomy and laceration	n (%)	2 (6.3)	0 (0.0)
Intact	n (%)	15 (46.9)	15 (45.5)
Frequency of PFMT			
Did not perform	n (%)	21 (65.6)	19 (57.6)
Performed sometimes	n (%)	9 (28.1)	14 (42.4)
Performed daily	n (%)	2 (6.3)	0 (0.0)
Daily number of PFMT performed			
0–9	n (%)	27 (84.4)	27 (81.8)
10–29	n (%)	5 (15.6)	5 (15.2)
More than 30	n (%)	0 (0.0)	1 (3.0)
Urinary incontinence			
Continent	n (%)	19 (59.4)	19 (57.6)
Incontinent	n (%)	13 (40.6)	14 (42.4)
ICIQ-SF score	M (SD)	2.81 (3.85)	2.91 (3.99)

*VP* vaginal palpation, *TAU* transabdominal ultrasound, *SD* standard deviation, *PFMT* pelvic floor muscle training, *ICIQ-SF* International Consultation on Incontinence Questionnaire-Short Form

### Outcome measures main analysis

The mean ± standard deviation (SD) bladder base displacement before the intervention was 5.80 ± 4.69 mm in the VP group and 6.04 ± 5.05 mm in the TAU group, which were not significantly different. After the intervention, the bladder base displacement was 6.91 ± 3.31 mm in the VP group and 6.19 ± 4.05 in the TAU group. The change in bladder base displacement was greater in the VP group (1.11 ± 2.34 mm) than in the TAU group (0.15 ± 3.28 mm); however, the difference was not statistically significant (Table 2).

**Table 2:** Changes in bladder base displacement

	VP (n = 32)		TAU (n = 33)		*t*-value	*p*-value
	Mean	SD	Mean	SD		
Displacement (mm)						
Pre	5.80	4.69	6.04	5.05		
Post	6.91	3.31	6.19	4.05		
Change	1.11	2.34	0.15	3.28	1.355	0.181

*VP* vaginal palpation, *TAU* transabdominal ultrasound, *SD* standard deviation

In the two-item questionnaire to assess the understanding of PFM contraction, the question ‘Do you know where to contract in the body?’ was answered as ‘Hard to understand’ by nine participants and ‘Did not understand’ by one participant in the VP group before the intervention. The question ‘Do you understand the sensation of contracting the pelvic floor muscles?’ was answered with ‘Hard to understand’ by eight participants and ‘Did not understand’ by two participants, in the VP group before the intervention. After the intervention, both items of the questionnaire were answered by all 32 participants in the VP group as either ‘Extremely well’ or ‘Moderately well’. In contrast, the first question was answered with ‘Hard to understand’ and ‘Did not understand’ by five and two participants in the TAU group, respectively, before the intervention. The second question was answered with ‘Hard to understand’ by eight participants in this group. After the intervention, in the TAU group, two participants answered the first question with ‘Hard to understand’, one of whom also answered the second question with ‘Hard to understand’. Nevertheless, there was no statistically significant difference between the two groups regarding the changes in the understanding of PFM contraction after implementation of the two teaching methods (Table 3).

**Table 3:** Change in understanding PFM contraction

	VP (n = 32)		TAU (n = 33)		*t*-value	*p*-value
	Mean	SD	Mean	SD		
Understanding of PFMC (score)						
Pre	6.59	2.09	7.12	1.83		
Post	9.43	0.88	9.09	1.26		
Change	2.84	1.97	1.97	1.63	1.946	0.056

*PFM* pelvic floor muscle, *PFMC* pelvic floor muscle contraction, *VP* vaginal palpation, *TAU* transabdominal ultrasound, *SD* standard deviation

### Ancillary analysis

Participants with perineal injuries or urinary incontinence were sub-grouped and analysed to determine the differences in the results according to the teaching method.

Participants in the VP and TAU groups were subdivided into two sub-groups according to the presence or absence of perineal injuries (episiotomy or laceration)—the perineal injury group and perineal intact group (Table 4). Seventeen participants in the VP group and 18 in the TAU group had perineal injuries. Women who had perineal injuries (episiotomy or laceration) received sutures for the same. Pre-test bladder base displacement was similar between the groups. Before and after the intervention, bladder floor displacement was larger in the perineal intact group than in the perineal injury group. Specifically, the change in bladder floor displacement after the intervention in participants in the VP group with perineal injuries was 1.55 ± 2.71 mm, while that in participants in the TAU group with injuries was − 0.10 ± 4.08 mm, which were not statistically significantly different. Furthermore, the change in women without injuries (perineal intact group) was 0.61 ± 1.80 mm in the VP group and 0.46 ± 2.02 mm in the TAU group, with no statistically significant differences between the two groups.

**Table 4:** Changes in displacement of the bladder base according to the perineal condition

	Perineal injury (n = 35)				*t* value	*p* value	Perineal intact (n = 30)				*t* value	*p* value
	VP (n = 17)		TAU (n = 18)				VP (n = 15)		TAU (n = 15)			
	Mean	SD	Mean	SD			Mean	SD	Mean	SD		
Displacement (mm)												
Pre	4.83	4.23	4.91	5.15			6.90	5.07	7.40	4.75		
Post	6.38	2.69	4.81	3.93			7.51	3.91	7.86	3.66		
Change	1.55	2.71	− 0.10	4.08	− 1.171	0.252	0.61	1.80	0.46	2.02	0.507	0.616

*VP* vaginal palpation, *TAU* transabdominal ultrasound, *SD* standard deviation

Similarly, participants in the VP and TAU groups were subdivided into the incontinent and continent group depending on the presence or absence of urinary incontinence, respectively (Table 5). Thirteen participants (40.6%) in the VP group and 14 (42.4%) in the TAU group had urinary incontinence. Bladder base displacement before the intervention was comparable between the groups. Unexpectedly, both before and after the intervention, the bladder base displacement was larger in the incontinent group than in the continent group. Specifically, the change in bladder floor displacement after the intervention in participants in the incontinent group was 0.75 ± 2.09 mm in the VP group and − 0.95 ± 2.93 mm in the TAU group with no statistically significant differences between the two groups. Furthermore, the change in bladder floor displacement after the intervention in participants in the continent group was 1.35 ± 2.52 mm and 0.96 ± 3.35 mm in the VP and TAU groups, respectively, although no statistically significant differences were observed between the two groups.

**Table 5:** Changes in displacement of the bladder base according to urinary incontinence

	Incontinent (n = 27)				*t* value	*p* value	Continent (n = 38)				*t* value	*p* value
	VP (n = 13)		TAU (n = 14)				VP (n = 19)		TAU (n = 19)			
	Mean	SD	Mean	SD			Mean	SD	Mean	SD		
Displacement (mm)												
Pre	6.47	5.57	8.05	5.87			5.34	4.07	4.57	3.88		
Post	7.22	4.07	7.10	3.82			6.69	2.78	5.53	4.19		
Change	0.75	2.09	− 0.95	2.93	1.747	0.094	1.35	2.52	0.96	3.35	0.400	0.692

*VP* vaginal palpation, *TAU* transabdominal ultrasound, *SD* standard deviation

### Adverse events

No adverse events associated with the two interventions were reported by the participants.

## Discussion

This study examined the changes in comprehension of PFM contraction by comparing two teaching methods, VP and TAU, in women after vaginal delivery. Contrary to our hypothesis that VP would be more suitable than TAU in improving the understanding of PFM contraction, we found no significant differences between the two teaching methods.

The main difference between the two interventions is that VP has a more direct effect than TAU, which is device-based. Although the changes in PFM contraction after the intervention were greater in the VP group than in the TAU group, no statistically significant differences were observed between the two groups. The lack of such differences could be explained by the fact that the feedback received by the participants involved physical sensations (tactile and visual) in both interventions. Providing feedback to the participant during the intervention was shown to be effective in PFM training [24]. Our findings suggested that both tactile and visual feedback may be useful and easy to understand. Another reason for the non-significant differences may be the small sample size. The previous study on which we based our sample size calculations included a Western population with different body characteristics from those in Japanese women. Nevertheless, given the small sample size of our study, such a hypothesis may need to be tested in larger samples.

Sub-group analyses also indicated that in participants with perineal injuries or urinary incontinence, changes in bladder base displacement after the intervention were greater with VP than with TAU, although the differences were not statistically significant. Furthermore, the displacement of the bladder base was larger in the incontinent group than in the continent group. This result corresponds well with those of previous studies in which the inability to correctly perform PFM contraction was not associated with urinary incontinence [25].

VP has the advantage that it does not require expensive tools and can be performed at any time desired by the participant. The disadvantage is that it is invasive and participants may feel uncomfortable. In contrast, TAU is totally non-invasive, and the patient does not need to get undressed. The advantages of TAU may be used in populations where VP may not be appropriate, such as in women with vaginal pain or who feel uncomfortable [14]. Our results verified that TAU is as useful as VP as a teaching method in postpartum women. However, TAU requires a full bladder, which may be difficult in women with reduced bladder function capacity or bladder urgency, and it may be difficult to obtain a clear image in women with dense abdominal scar tissue [13]. An additional disadvantage of using TAU alone in postpartum women is that it does not allow easy observation of the vulva. Therefore, the vulva should be monitored for perineal injuries.

Within the groups, bladder base displacement was larger after the intervention than before it. In order to increase the muscle strength, a previous study recommended the use of a training protocol that follows strength-training principles, emphasises close to maximum contractions, and lasts at least 8 weeks [26]. Therefore, the observed improvement in our study may not be attributed to increased PFM strength after the intervention due to the short duration of the intervention but rather can be attributed to changes in the comprehension of PFM contraction through the intervention.

A previous systematic review reported that TAU measures bladder base displacement between 3.7 and 8.7 mm in the supine position [27]. A recent study measured bladder base displacement in the midsagittal plane using TAU and reported PFM displacement of 7.8 ± 4.5 mm in the supine position in 17 young healthy nulliparous women [28]. In our study, the participants were low-risk women, predominantly multiparous, and at 4–6 weeks after delivery. The displacement of the bladder base during PFM contraction was not comparable to that in young healthy nulliparous women but was not significantly inferior. Although different results may be observed in participants with pelvic floor disorders, our results might be useful when targeting low-risk postpartum women.

### Limitations

This study had a few limitations. First, it was not possible to mask the group allocation. Second, we did not investigate whether the intervention was being implemented continuously as we compared the changes in the participant’s comprehension of PFM contraction with each teaching method. Future research is needed to evaluate the study outcomes after continuing PFM training for a longer duration. Finally, since the participants were low-risk postpartum women, it is necessary to also consider high-risk participants in the future.

## Conclusions

In this study, we hypothesised that VP is better than TAU for improving comprehension of PFM contraction as assessed by bladder base displacement during PFM contraction and the changes in the understanding of PFM contraction after the intervention. However, we found no significant differences between these methods, thus suggesting that both teaching methods might be useful for the comprehension of PFM contraction after vaginal delivery. In clinical practice, individual preferences must be considered, and a personalised teaching method should be chosen according to the various advantages and disadvantages of VP and TAU.

## Data Availability

The datasets generated during and/or analysed during the current study are available from the corresponding author on reasonable request.

## Abbreviations

CI: Confidence interval
ICIQ-SF: International Consultation on Incontinence Questionnaire-Short Form
PFM: Pelvic floor muscle
SD: Standard deviation
TAU: Transabdominal ultrasound
VP: Vaginal palpation
